# Supplementary material for: Discovery of Phenylcarbamoylazinane-1,2,4-Triazole Amides Derivatives as the Potential Inhibitors of Aldo-Keto Reductases (AKR1B1 & AKRB10): Potential Lead Molecules for Treatment of Colon Cancer
Source: Molecules. 2022 Jun 21;27(13):3981. doi: 10.3390/molecules27133981 (PMC9268700; doi:10.3390/molecules27133981)
Supplement: Supplementary file 1 [file molecules-27-03981-s001.zip › molecules-1707883-supplementary.pdf]

Supplementary Materials

Discovery of Phenylcarbamoylazinane-1,2,4-triazole Amides Derivatives as the Potential Inhibitors of Aldo-Keto Reductases (AKR1B1 & AKRB10): Potential Lead Molecules for Treatment of Colon Cancer

Amna Saeed[1], Syeda Abida Ejaz[1*], Muhammad Sarfraz[2], Nissren Tamam[3], Farhan Siddique[4,5], Naheed Riaz[6,] Faizan Abul Qais[7], Samir Chtita[8] and Jamshed Iqbal[9*]

[1] Department of Pharmaceutical Chemistry, Faculty of Pharmacy, The Islamia University of Bahawalpur, Bahawalpur, Pakistan, 63100
[2] College of Pharmacy, Al Ain University, Al Ain, Postal code 64141, United Arab Emirates
[3] Department of physics, College of Science, Princess Nourah bint Abdulrahman University, P.O Box 84428,Riyadh 11671,Saudi Arabia
[4] Department of Chemistry and Biochemistry, Texas Tech University, Lubbock, USA, TX 79409-1061
[5] Department of Pharmacy, Royal Institute of Medical Sciences (RIMS) Multan, Pakistan, 60000
[6] Department of Chemistry, Baghdad-ul-Jadeed Campus, The Islamia University of Bahawalpur, Bahawalpur, Pakistan, 63100
[7] Department of Agricultural Microbiology, Faculty of Agricultural Sciences, Aligarh Muslim University, Aligarh, India, UP 202002
[8] Laboratory of Analytical and Molecular Chemistry, Faculty of Sciences Ben M′Sik, , Hassan II University of Casablanca, BP7955 Sidi Othmane, Casablanca, Morocco.
[9]Centre for Advanced Drug Research, COMSATS University Islamabad, Abbottabad Campus, Abbotabad, Pakistan, 22060

*Corresponding Authors:
Syeda Abida Ejaz; abida.ejaz@iub.edu.pk
Jamshed Iqbal; jamshediqb@gmail.com
To whom correspondence should address:

Dr. Syeda Abida Ejaz; *Department of Pharmaceutical Chemistry, Faculty of Pharmacy, The Islamia University of Bahawalpur, Pakistan.*, Postal Code 63100, Pakistan Tel: +92-062-9250245 Fax: +92-062-9250245, E-Mail: abida.ejaz@iub.edu.pk; abidaejaz2010@gmail.com

3. Results and discussion

3.2. Density Functional Theory (DFTs)

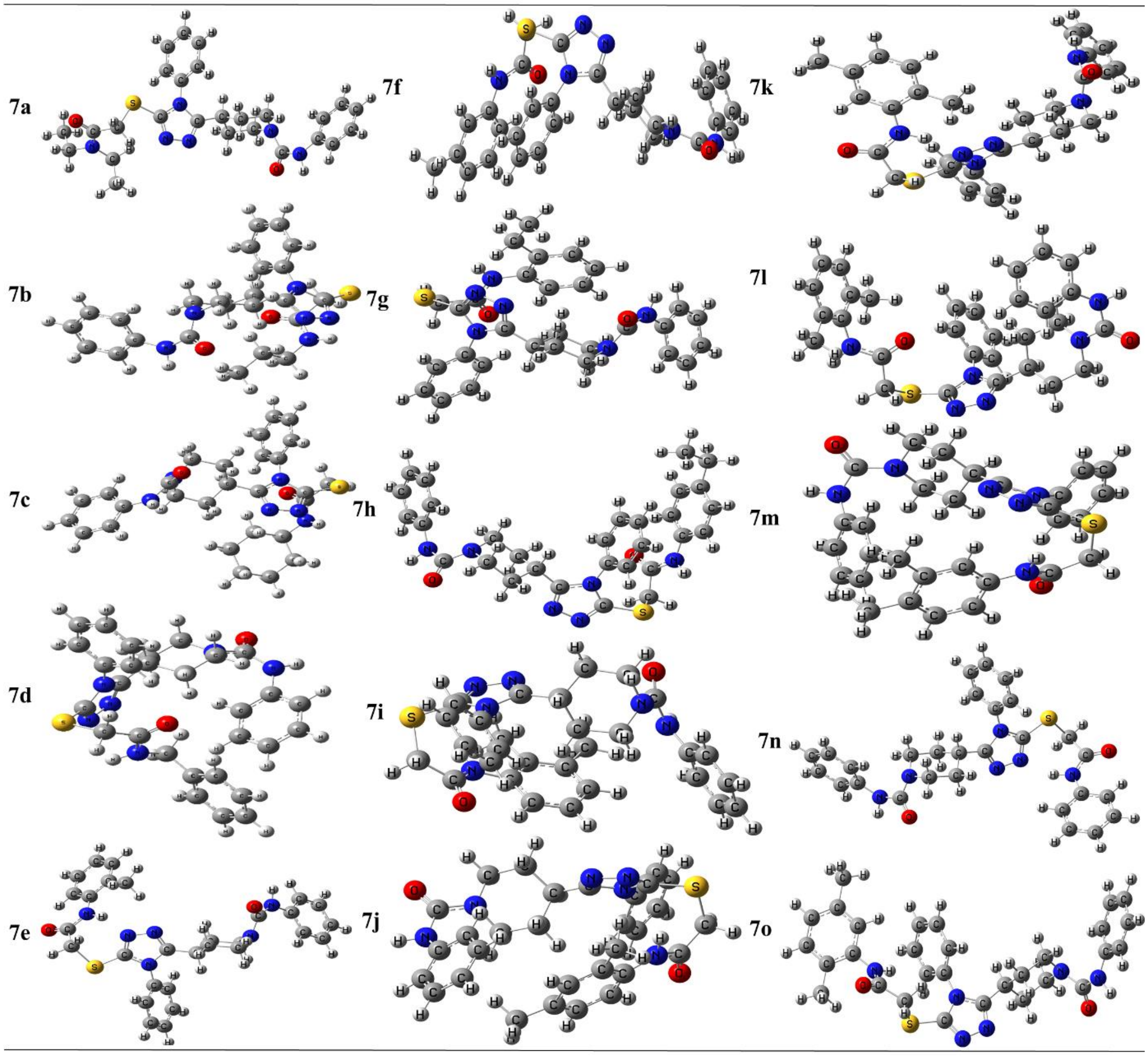

Figure S1: Optimized structures of phenylcarbamoylazinane-1,2,4-triazole amides derivatives in gas phase

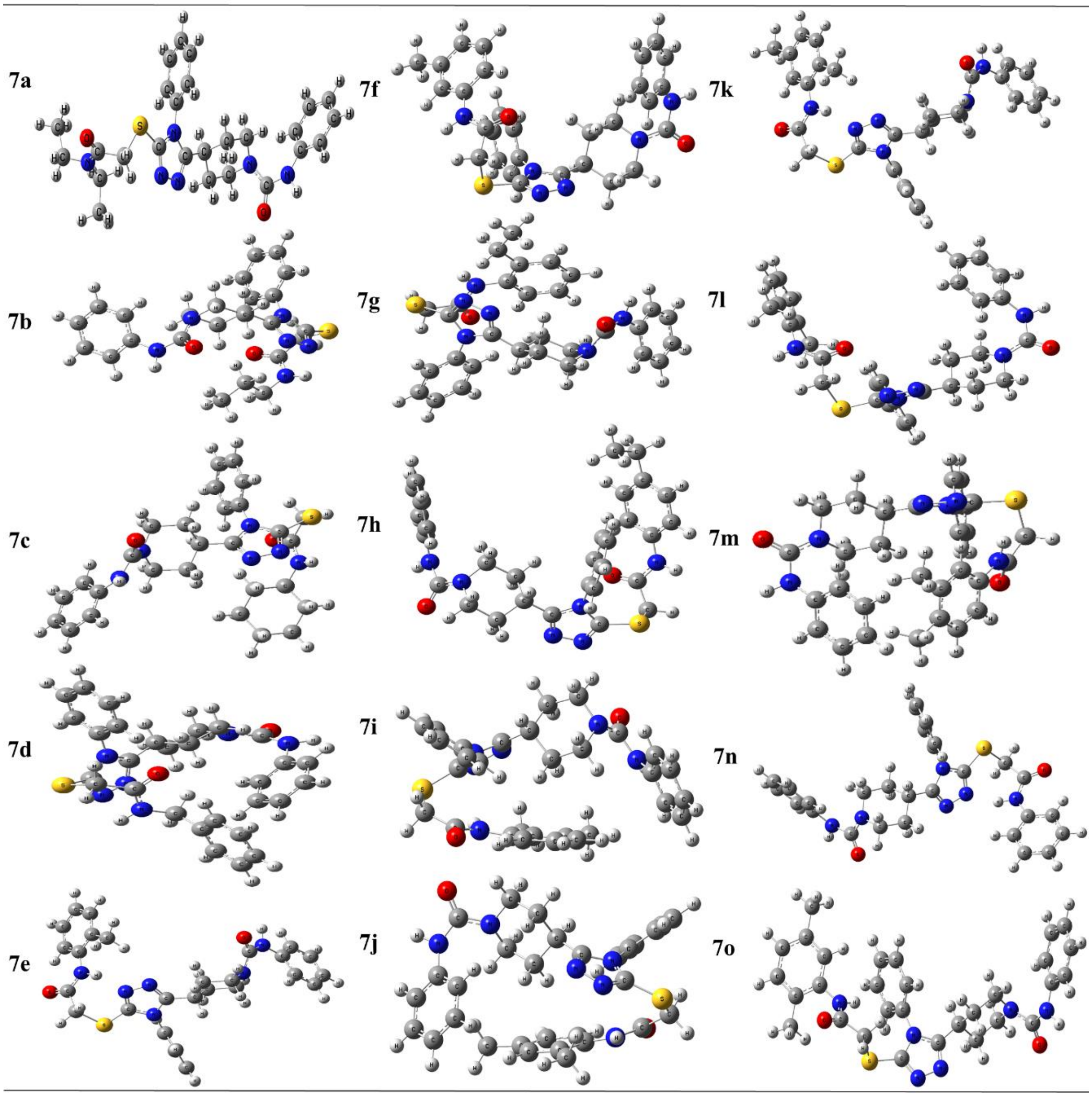

Figure S2: Optimized structures of phenylcarbamoylazinane-1,2,4-triazole amides derivatives in solvent phase (ethanol)

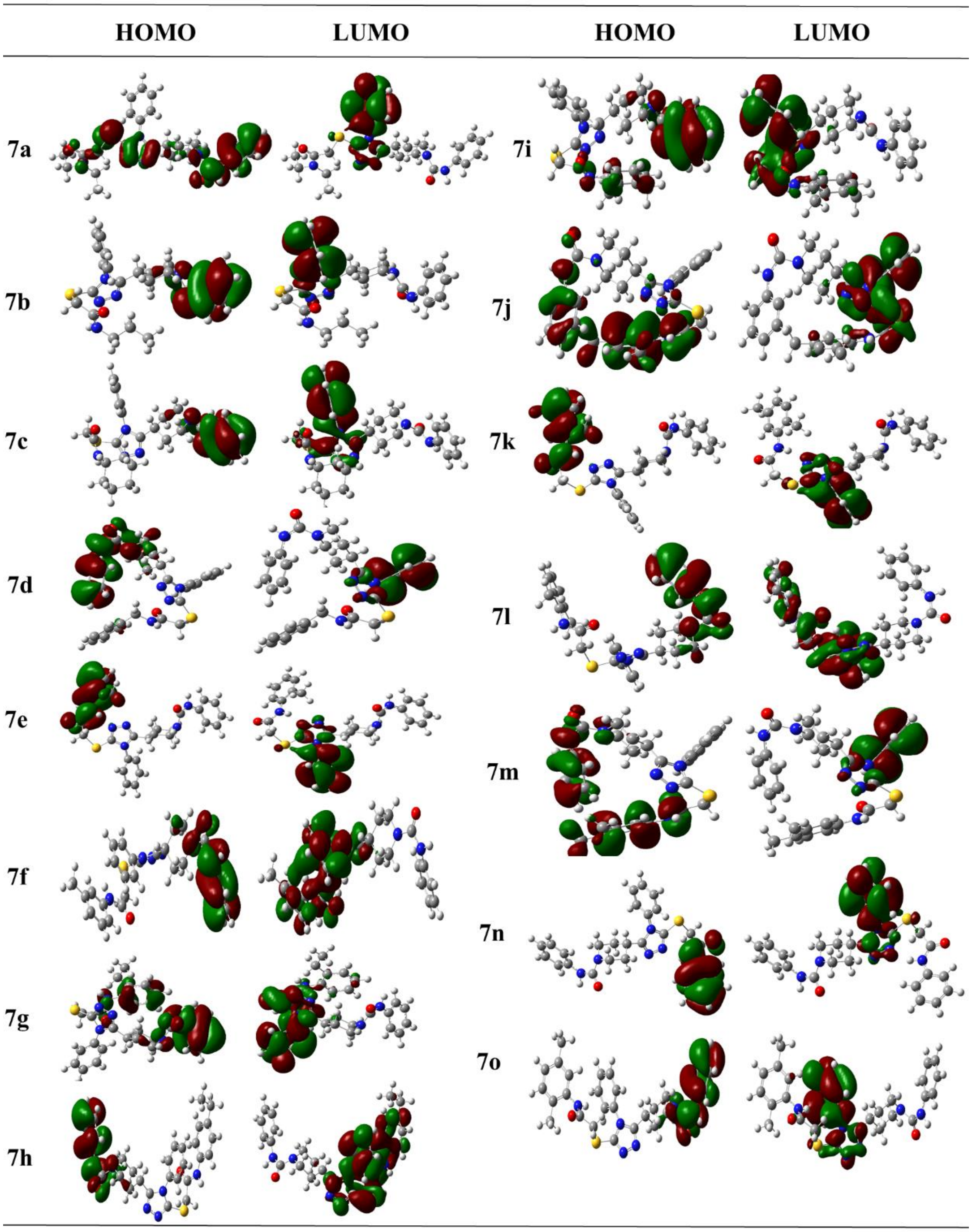

Figure S3: HOMO LUMO structures of phenylcarbamoylazinane-1,2,4-triazole amides derivatives in gas phase

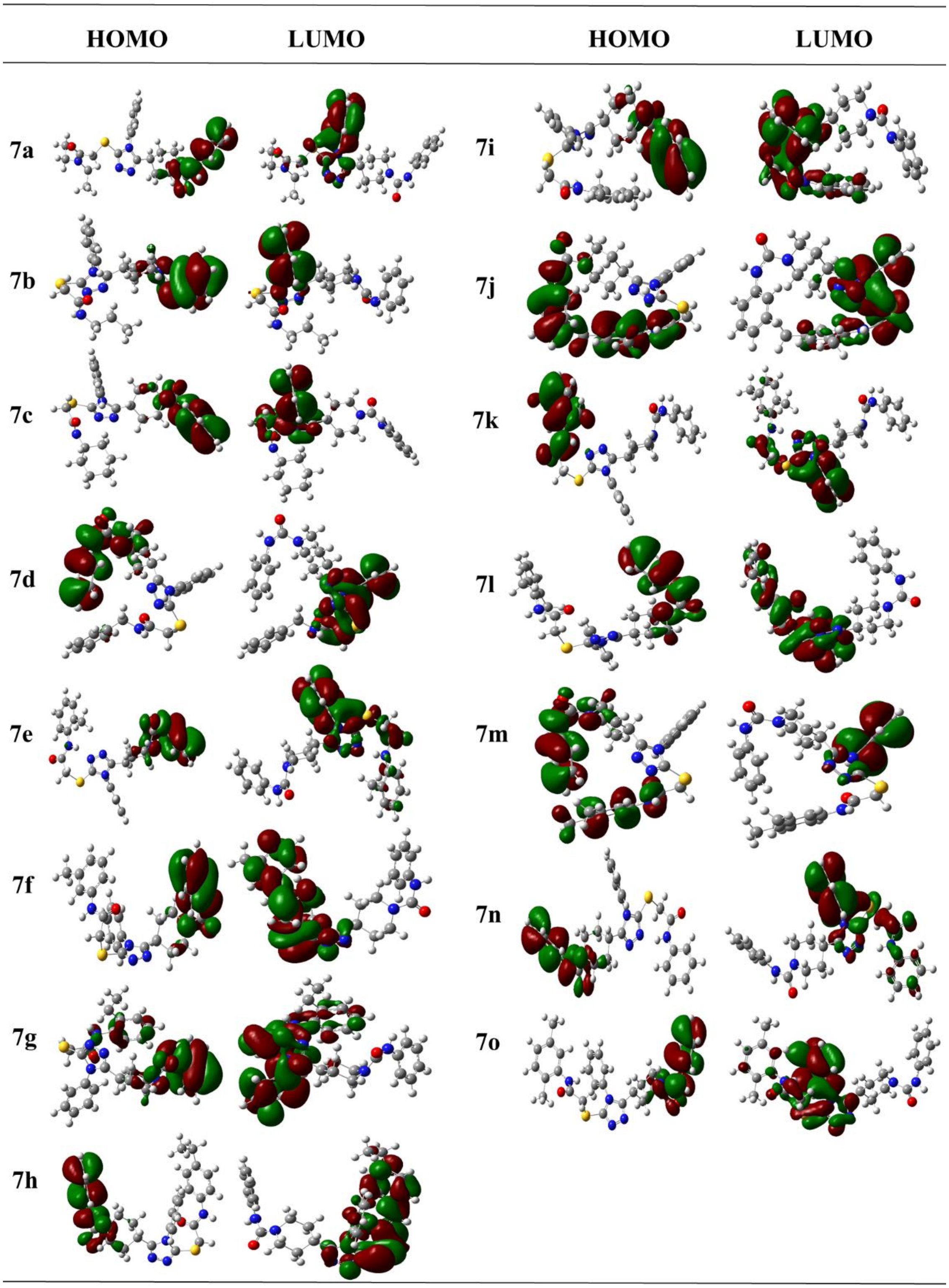

Figure S4: HOMO LUMO structures of phenylcarbamoylazinane-1,2,4-triazole amides derivatives in solvent phase (ethanol)

Table S1. Energetic parameters of phenylcarbamoylazinane-1,2,4-triazole amides derivatives (**7a-7o**) in gas phase

Compound	E_{HOMO} (eV)	E_{LUMO} (eV)	ΔE_{gap} (eV)	Potential Ionization I(eV)	Affinity A(eV)
**7a**	-0.225	-0.045	0.180	0.225	0.045
**7b**	-0.225	-0.041	0.184	0.225	0.041
**7c**	-0.224	-0.042	0.182	0.224	0.042
**7d**	-0.223	-0.040	0.184	0.223	0.040
**7e**	-0.209	-0.052	0.158	0.209	0.052
**7f**	-0.223	-0.046	0.176	0.223	0.046
**7g**	-0.221	-0.046	0.175	0.221	0.046
**7h**	-0.222	-0.045	0.177	0.222	0.045
**7i**	-0.223	-0.046	0.177	0.223	0.046
**7j**	-0.221	-0.049	0.173	0.221	0.049
**7k**	-0.207	-0.052	0.155	0.207	0.052
**7l**	-0.220	-0.035	0.185	0.220	0.035
**7m**	-0.221	-0.044	0.177	0.221	0.044
**7n**	-0.212	-0.052	0.160	0.212	0.052
**7o**	-0.227	-0.059	0.168	0.227	0.059

Table S2. Energetic parameters of phenylcarbamoylazinane-1,2,4-triazole amides derivatives (**7a-7o**) in solvent phase (ethanol)

**Compound**	**E_{HOMO} (eV)**	**E_{LUMO} (eV)**	**ΔE_{gap} (eV)**	**Potential Ionization I(eV)**	**Affinity A(eV)**
**7a**	-0.225	-0.037	0.188	0.225	0.037
**7b**	-0.225	-0.035	0.190	0.225	0.035
**7c**	-0.224	-0.039	0.186	0.224	0.039
**7d**	-0.227	-0.034	0.193	0.227	0.034
**7e**	-0.225	-0.039	0.185	0.225	0.039
**7f**	-0.226	-0.044	0.182	0.226	0.044
**7g**	-0.224	-0.044	0.180	0.224	0.044
**7h**	-0.224	-0.043	0.181	0.224	0.043
**7i**	-0.226	-0.043	0.182	0.226	0.043
**7j**	-0.223	-0.045	0.178	0.223	0.045
**7k**	-0.222	-0.039	0.183	0.222	0.039
**7l**	-0.226	-0.035	0.191	0.226	0.035
**7m**	-0.225	-0.035	0.190	0.225	0.035
**7n**	-0.225	-0.039	0.186	0.225	0.039
**7o**	-0.225	-0.046	0.179	0.225	0.046

Table S3. Quantum chemical descriptors of phenylcarbamoylazinane-1,2,4-triazole amides derivatives (**7a-7o**) in gas phase

Compound	Hardness (η)	Softness (S)	Electronegativity (X)	Chemical Potential (μ)	Electrophilicity Index (ω)
**7a**	0.090	5.57	0.135	-0.135	0.102
**7b**	0.092	5.45	0.133	-0.133	0.097
**7c**	0.091	5.50	0.133	-0.133	0.097
**7d**	0.092	5.45	0.132	-0.132	0.094
**7e**	0.079	6.34	0.130	-0.130	0.108
**7f**	0.088	5.67	0.134	-0.134	0.102
**7g**	0.088	5.71	0.134	-0.134	0.102
**7h**	0.088	5.65	0.134	-0.134	0.101
**7i**	0.088	5.66	0.135	-0.135	0.103
**7j**	0.086	5.78	0.135	-0.135	0.105
**7k**	0.077	6.47	0.129	-0.129	0.108
**7l**	0.092	5.41	0.128	-0.128	0.088
**7m**	0.089	5.64	0.132	-0.132	0.099
**7n**	0.080	6.24	0.132	-0.132	0.109
**7o**	0.084	5.96	0.143	-0.143	0.122

Compound	Electrodonating power (ω^-)	Electroaccepting power (ω^+)	Net Electrophilicity ($\Delta\omega^\pm$)
**7a**	0.180	0.045	0.225
**7b**	0.174	0.041	0.215
**7c**	0.175	0.042	0.217
**7d**	0.172	0.040	0.212
**7e**	0.184	0.053	0.237
**7f**	0.181	0.046	0.227
**7g**	0.180	0.046	0.226
**7h**	0.179	0.045	0.224
**7i**	0.181	0.046	0.227
**7j**	0.184	0.049	0.233
**7k**	0.183	0.053	0.236
**7l**	0.163	0.036	0.199
**7m**	0.177	0.044	0.221
**7n**	0.185	0.053	0.238
**7o**	0.204	0.061	0.264

Table S4. Quantum chemical descriptors of phenylcarbamoylazinane-1,2,4-triazole amides derivatives (**7a-7o**) in solvent phase (ethanol)

Compound	Hardness (η)	Softness (S)	Electronegativity (X)	Chemical Potential (μ)	Electrophilicity Index (ω)
**7a**	0.094	5.33	0.131	-0.131	0.091
**7b**	0.095	5.25	0.130	-0.130	0.089
**7c**	0.093	5.38	0.131	-0.131	0.093
**7d**	0.097	5.17	0.131	-0.131	0.088
**7e**	0.093	5.40	0.132	-0.132	0.094
**7f**	0.091	5.49	0.135	-0.135	0.100
**7g**	0.090	5.55	0.134	-0.134	0.100
**7h**	0.091	5.52	0.134	-0.134	0.099
**7i**	0.091	5.49	0.134	-0.134	0.099
**7j**	0.089	5.61	0.134	-0.134	0.101
**7k**	0.091	5.47	0.131	-0.131	0.093
**7l**	0.095	5.24	0.130	-0.130	0.089
**7m**	0.095	5.27	0.130	-0.130	0.090
**7n**	0.093	5.38	0.132	-0.132	0.093
**7o**	0.089	5.60	0.135	-0.135	0.103

Compound	Electrodonating power (ω^-)	Electroaccepting power (ω^+)	Net Electrophilicity ($\Delta\omega^{\pm}$)
**7a**	0.169	0.038	0.206
**7b**	0.166	0.036	0.202
**7c**	0.171	0.039	0.210
**7d**	0.166	0.035	0.201
**7e**	0.171	0.039	0.211
**7f**	0.179	0.044	0.223
**7g**	0.178	0.044	0.222
**7h**	0.177	0.043	0.220
**7i**	0.178	0.043	0.221
**7j**	0.179	0.045	0.224
**7k**	0.170	0.039	0.209
**7l**	0.166	0.036	0.202
**7m**	0.166	0.036	0.202
**7n**	0.171	0.039	0.211
**7o**	0.182	0.046	0.228

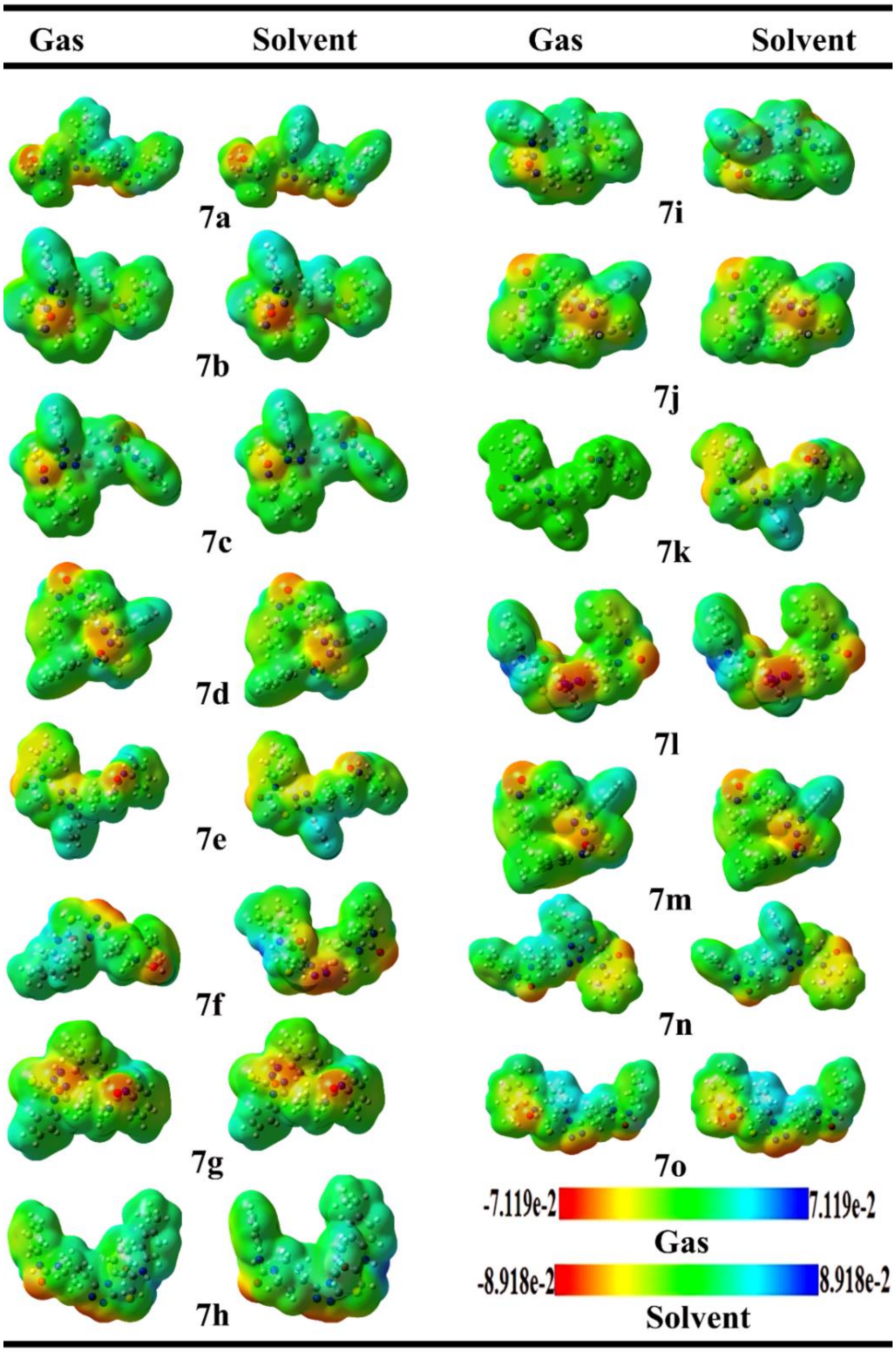

Figure S5: Electrostatic potential (ESP) of phenylcarbamoylazinane-1,2,4-triazole amides derivatives

3.3. Molecular docking

3.3.1. Detailed molecular docking discussion of protein AKR1B1

3D and 2D conformations of the potent derivatives are shown in **Figure S6** and **S7**, respectively. The interactions were observed as strong hydrogen bonding, van der Waals interactions, π-interactions which includes π-π, π-cation and π-anion interactions. Only one hydrogen bond interaction was formed by oxygen of methyl acetate group and oxygen of (3,S)Octahydro-1H-indole-1-carbaldehyde of **vincristine** with amino acid residue Arg268. Electrostatic interaction (π-cation) was formed between benzene ring of 6-methoxyindoline of **vincristine** and Arg268.

Hydrogen bond complex was observed by oxygen of trimethyl phosphate, oxygen of benzamide, hydroxyl group of dimethyl tetrahydrofuran-3, 4-diol of **NAP** with amino acid residue Lys262. One of the hydrogen bonds interactions was formed between oxygen of methyl dihydrogen phosphate of **NAP** and Lys21. Oxygen of trimethyl tetrahydrofuran-3-ol of **NAP** exhibited hydrogen bonding with Arg268. Hydrogen bond interaction was also formed between hydroxyl group of dimethyl tetrahydrofuran-3, 4-diol of **NAP** and Arg217. Strong hydrophobic interaction (π-sigma) was seen between Pyrimidin-4-amine group of **NAP** and Leu228. Electrostatic interaction (π-cation) was observed by Phosphorous of methyl dihydrogen phosphate of **NAP** with Asp216.

Hydrogen bond complex was formed by hydrogen of N-phenylacetamide of ligand **7e** with amino acid residue of active pocket Ile260. Another hydrogen bonding was observed between oxygen of N-phenylacetamide of ligand **7e** and Leu212. 2-mercapto-N-(*o*-tolyl) acetamide of ligand **7e** formed two hydrogen bond complexes, one with Asp216 and second with Arg268. One carbon hydrogen bond found between Pro261 and oxygen of N-phenylacetamide of ligand **7e**. Electrostatic interaction (π-cation) made by phenyl ring of ligand **7e** and Arg268. Another electrostatic interaction (π-anion) formed between Asp216 and toluene ring of ligand **7e**.

Oxygen of N-phenylacetamide of ligand **7f** built hydrogen bond complex with Leu212 and Ser214. The active site of AKR1B1 comprised amino acid Lys262 which formed hydrogen bonding with nitrogen of triazole group and oxygen of 2-mercapto-N-(*m*-tolyl) acetamide of ligand **7f**. Sulfur of 2-mercapto-N-(*m*-tolyl) acetamide of ligand **7f** made hydrogen bonding complex with amino acid residue Thr265. Nitrogen of triazole group of ligand **7f** created carbon

hydrogen bond interaction with Ser263. Amino acid residue Asp216 formed electrostatic interaction (π-anion) with triazole group and other with xylene of ligand **7f**. One of the electrostatic interactions (π-cation) was found between phenyl ring of **7f** with Arg268, other with xylene ring of ligand **7f** and Lys262.

Nitrogen of triazole group, sulfur of N-(4-ethylphenyl)-2-(methylthio) acetamide and oxygen of N-phenylacetamide of ligand **7h** were involved in making hydrogen bond interaction with Lys262. Oxygen of N-(4-ethylphenyl)-2-(methylthio) acetamide of ligand **7h** formed hydrogen bond complex with Ser214 and Leu212. Electrostatic interaction (π-cation) was formed by phenyl ring of ligand **7h** with Arg268.

Amino acid residue Lys262 formed hydrogen bonding interaction with sulfur of N-(2, 4-dimethylphenyl)-2(methylthio) acetamide of ligand **7j**. Hydrogen bonding was observed between oxygen of N-(2, 4-dimethylphenyl)-2(methylthio) acetamide of ligand **7j** and amino acid residue Ser214. Electrostatic interaction (π-anion) was formed by phenyl ring of ligand **7j** with Arg268. Another electrostatic interaction (π-cation) was formed by phenyl ring of N-phenylacetamide of ligand **7j** with Asp216.

Oxygen of N-phenylacetamide of ligand **7k** formed hydrogen bond complex with Leu212. Hydrogen of N-phenylacetamide of ligand **7k** formed hydrogen bonding with Ile260. Nitrogen of triazole group of ligand **7k** involved in hydrogen bonding with Lys262. Sulfur of N-(2, 4-dimethylphenyl)-2-(methylthio) acetamide of ligand **7k** involved in hydrogen bonding with Arg268 and Thr26. The electrostatic interaction (π-anion) was formed by triazole ring of ligand **7k** with Lys262. Another electrostatic interaction (π-cation) was formed by 1,2,4-trimethylbenzene of ligand **7k** with Asp216.

Amino acid residues Arg268 and Thr265 were involved in making hydrogen bond interaction with sulfur of N-(3, 4-dimethylphenyl)-2(methylthio) acetamide of ligand **7m**. Nitrogen of triazole group of ligand **7m** formed hydrogen bond complex with Lys262. Amino acid residues Gly213, Ser214 and Leu212 formed hydrogen bonding with oxygen of N-phenylacetamide of ligand **7m**. Electrostatic interaction (π-cation) was formed between phenyl ring of **7m** and Arg268.

3.3.2. Detailed molecular docking discussion of protein AKR1B10

The 3D and 2D interactions of the potent derivatives are given in **Figure S8** and **S9**, respectively. The interactions were observed as strong hydrogen bonding, van der Waals interactions, π-interactions which includes π-π, π-cation and π-anion interactions.

Methyl-2-hydroxyacetate group of **vincristine** was observed building two hydrogen bond interactions with amino acid residues Ala221 and Pro219. Two electrostatic interactions (π-cation) were formed by amino acid residues Lys263 and Arg269 with indole of **vincristine**.

Hydrogen bond complex was observed between hydrogen of pyrimidine-4-amine of **NAP** and Pro216. Hydrogen bonding was also observed between oxygen of acetamide of **NAP** and Lys22. Another hydrogen bond complex was formed by hydroxyl group of dimethyl hydrogen phosphate of **NAP** with Val265.

The binding mode involving hydrogen bond interactions formed by oxygen of N-phenylacetamide of ligand **7d** with amino acid residues Ser215, Gly214 and Leu213. The other hydrogen bonding was formed between sulfur of N-benzyl-2(methylthio) acetamide of ligand **7d** and Arg269. The nitrogen of triazole group of ligand **7d** also formed hydrogen bonding with Lys263. Electrostatic interaction (π-cation) was formed between sulfur of N-benzyl-2(methylthio) acetamide of **7d** and Thr266.

Significant Hydrogen bonding was formed by nitrogen of triazole group of ligand **7e** with Ser215. Another predominant hydrogen bonding was formed between oxygen of 2-mercapto-N-(*o*-tolyl) acetamide of ligand **7e** and Tyr49. Hydrogen bonding was also formed between oxygen of N-phenylacetamide of ligand **7e** and Pro216. A favorable hydrophobic interaction (π-π T-shaped) was formed between triazole ring and toluene ring of ligand **7e** and Trp21. Electrostatic interaction (π-cation) was formed between Arg269 and phenyl ring of ligand **7e**. Another electrostatic interaction (π-cation) was formed between nitrogen of triazole group and Lys22. Electrostatic interaction (π-cation) was also formed between toluene ring and his111.

Oxygen of N-(4-ethylphenyl) acetamide of ligand **7h** formed hydrogen bond interaction with amino acid residue Lys22. Nitrogen of triazole group, sulfur of N-(4-ethylphenyl)-2-(methylthio) acetamide and oxygen of N-phenylacetamide of ligand **7h** were involved in making hydrogen bond interaction with Lys263. Electrostatic interaction (π-cation) was formed by phenyl ring of ligand **7h** with Arg269.

Pro212 built hydrogen bond complex with oxygen of N-(2, 4-dimethylphenyl) acetamide of ligand **7j**. Electrostatic interaction (π-cation) was formed by triazole ring, phenyl ring of N-phenylacetamide of ligand **7j** with Lys263. Another electrostatic interaction π-cation was also formed by phenyl ring of triazole of ligand **7j** with Arg269.

Hydrogen of N-phenylacetamide of ligand **7k** made hydrogen bond interaction with Asp217. Oxygen of N-phenylacetamide of ligand **7k** formed hydrogen bonding with Lys263. Nitrogen of triazole of compound **7k** involved in hydrogen bonding with Arg269. Electrostatic interaction (π-cation) was formed by triazole ring of ligand **7k** with Arg269. Another electrostatic interaction of π-anion was formed by 1,2,4-trimethylbenzene of compound **7k** with Glu230.

The amino acid residue Lys263 was observed creating hydrogen bond interaction with sulfur and oxygen of N-(3, 4-dimethylphenyl)-2(methylthio) acetamide of ligand **7m**. Oxygen of N-(3, 4-dimethylphenyl)-2(methylthio) acetamide of ligand **7m** created hydrogen bond complex with Pro212. Amino acid residues Lys263 and Arg269 were involved in making electrostatic interaction (π-cation). Electrostatic interaction (π-cation) was formed by phenyl ring of triazole of **7m** with Arg269.

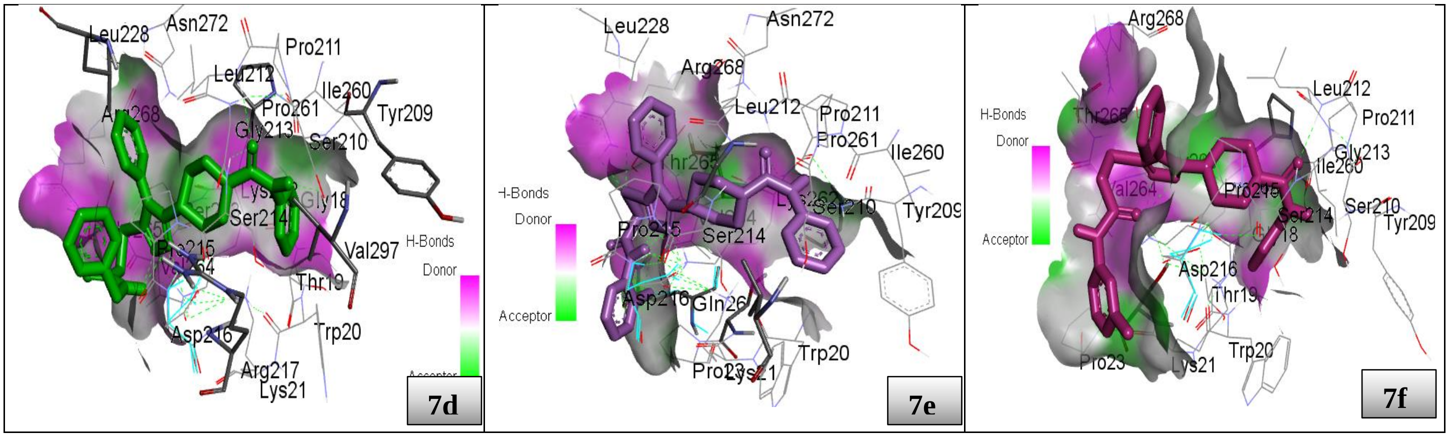

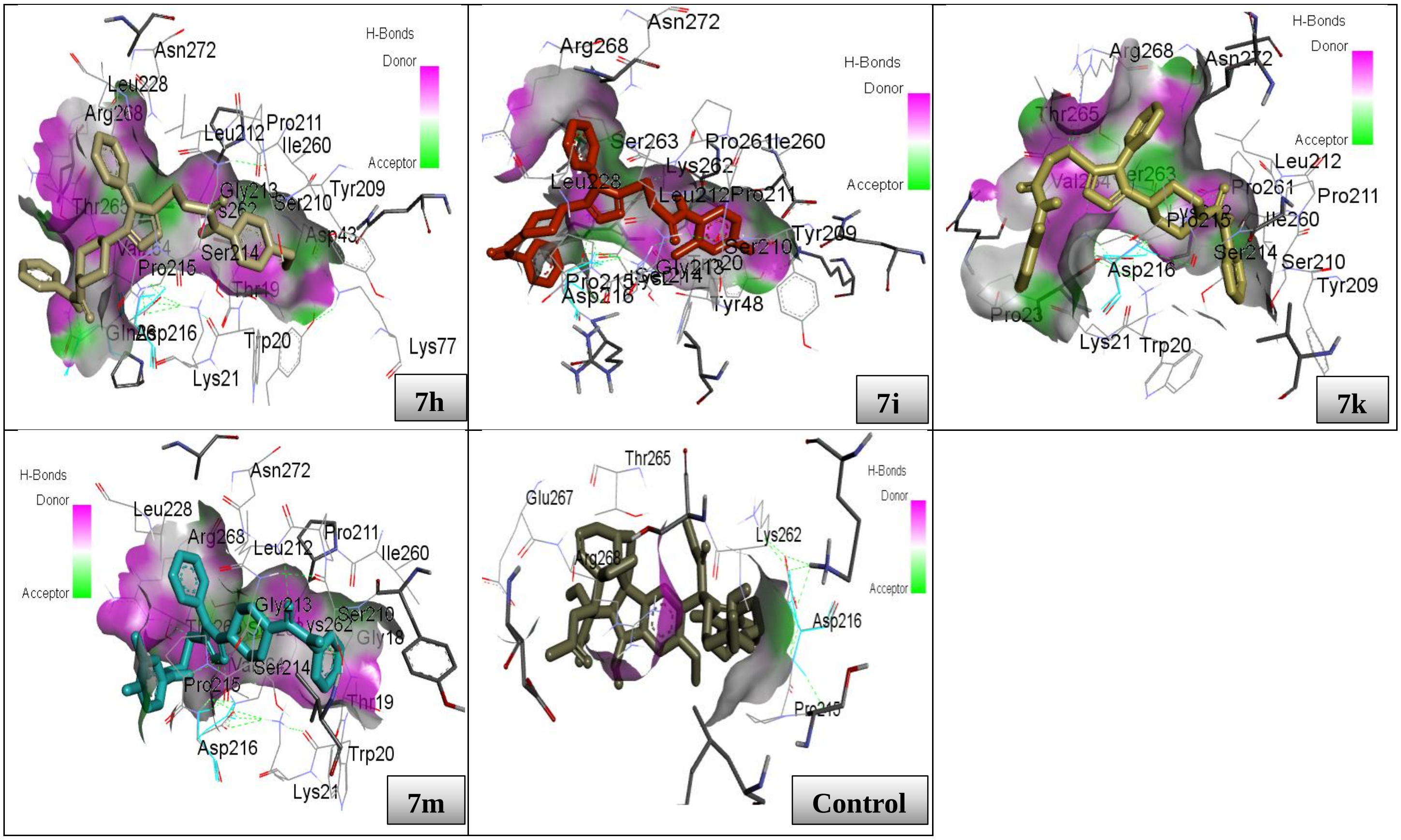

Figure S6. Showing 3D interactions of phenylcarbamoylazinane-1,2,4-triazole amides derivatives (**7a-o**) with active site of AKR1B1.

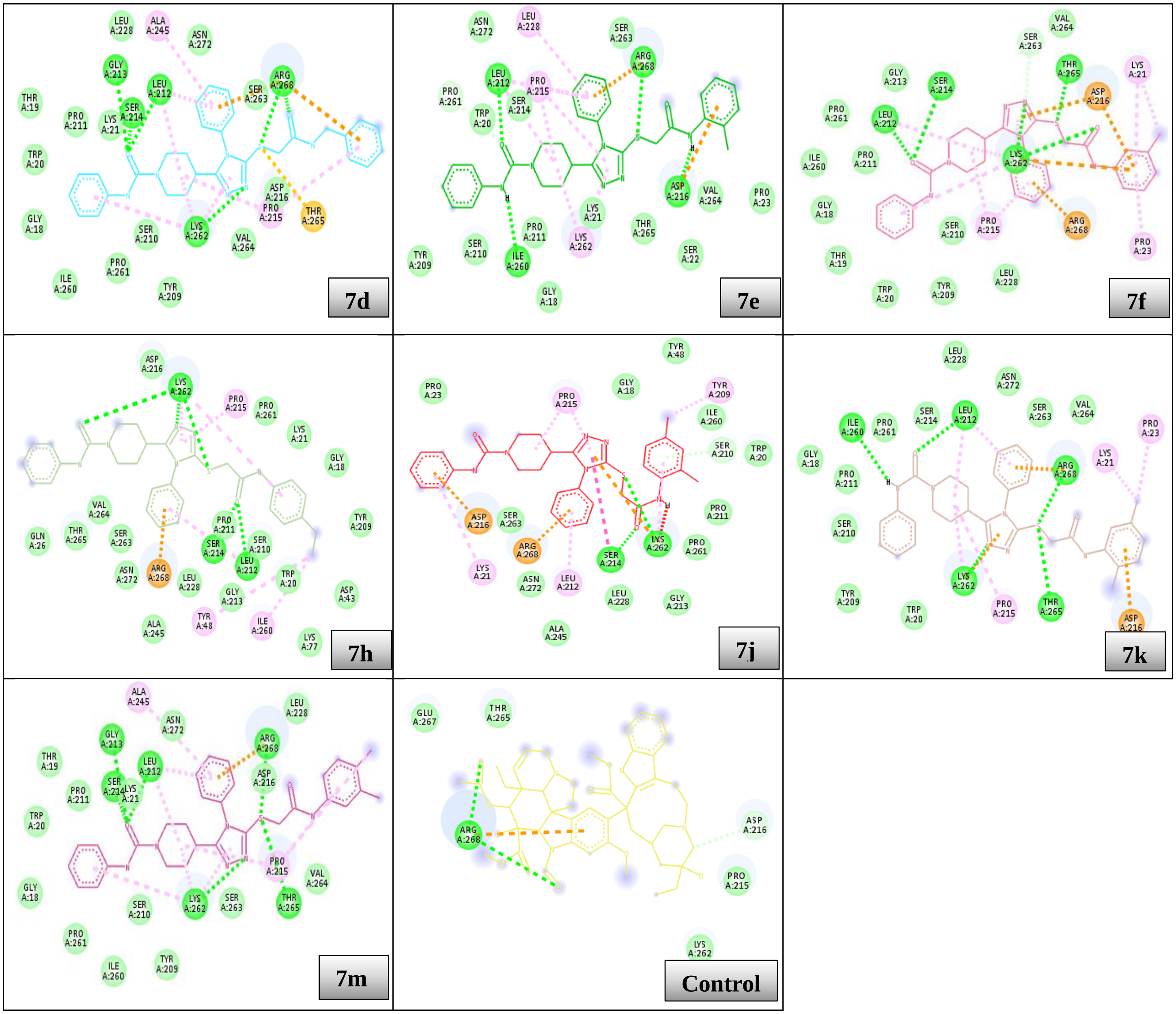

Figure S7. Showing 2D interactions of phenylcarbamoylazinane-1,2,4-triazole amides derivatives (**7a-o**) with active site of AKR1B1.

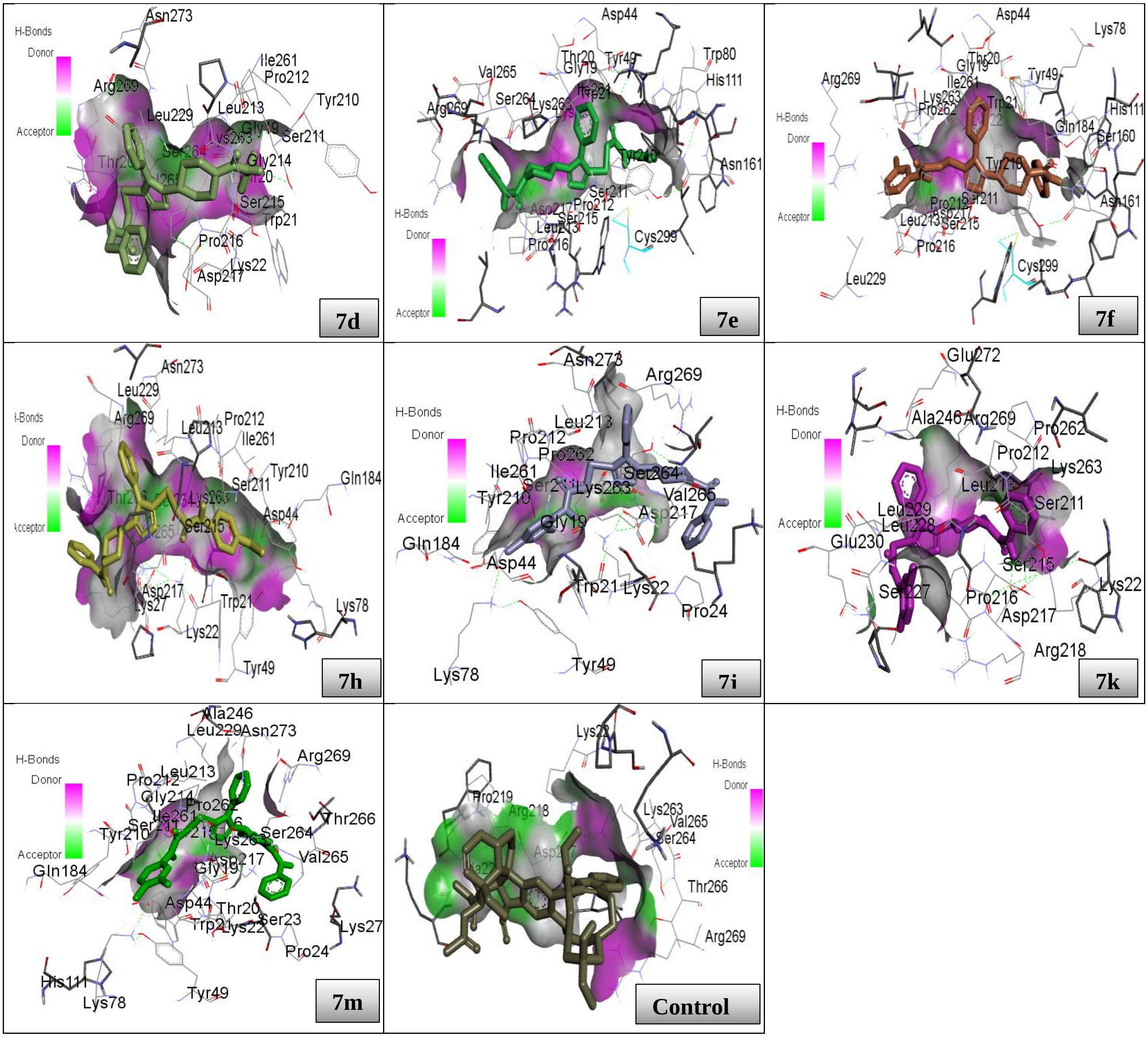

Figure S8. Showing 3D interactions of phenylcarbamoylazinane-1,2,4-triazole amides derivatives (**7a-o**) with active site of AKR1B10.

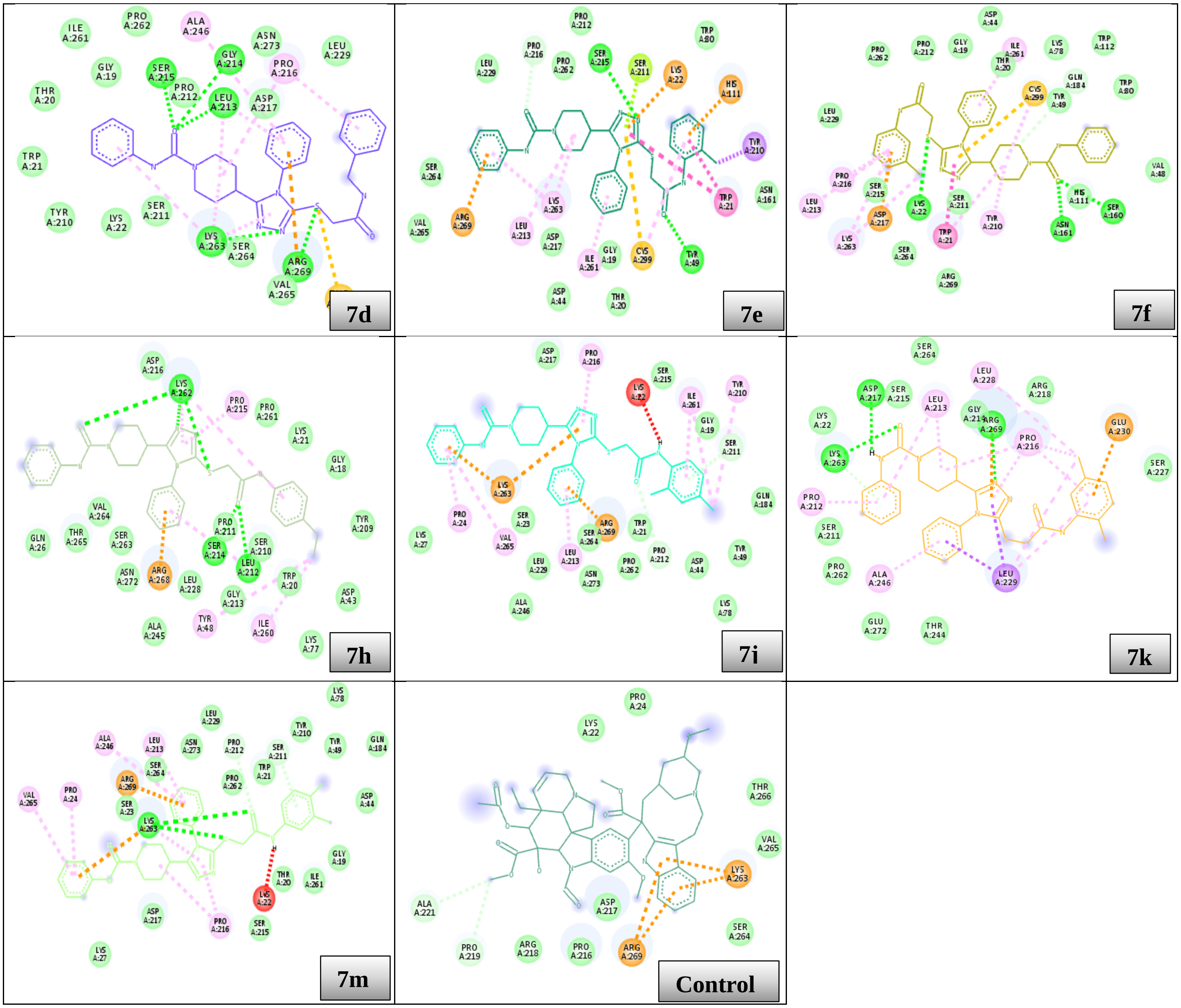

Figure S9. Showing 2D interactions of phenylcarbamoylazinane-1,2,4-triazole amides derivatives (**7a-o**) with active site of AKR1B10.

3.3.3. Docking with Nuclear factor kappa B (NF-κB)

NF-κB play a key function in various type of cancers where it promotes inflammation [1]. This has been demonstrated through the use of molecular docking with potent inhibitors (**7d** and **7f**). The results suggested that **7d** not only suppresses AKR1B1 but also NF-κB, which is the primary inflammatory factor. The detailed interactions of **7d** with NF-κB are shown in **Figure S10** below.

The amino acid residues of the active pocket of NF-κB are; Arg302, Lys310, Tyr306, His84, Met313, Arg171, Glu86, Ala88 and Lys87. Derivative **7d** had shown the minimum binding

energy of -22.12 kJ/mol interacting with one hydrogen bond and one π-aromatic bond. Amino acid residue Lys87 was observed creating hydrogen bond interaction with the acetamide moiety of **7d**. One π-aromatic interaction was observed between amino acid residue Lys87 and ethylbenzene of **7d**. Met313 and Ala88 were involved in van der Waals interaction.

Detailed binding interactions of other derivatives **7a** and **7f** with NF-κB are shown in F**igure S10** below. Derivative **7a** and **7f** had shown the binding energies of -21.48 kJ/mol and -20.92 kJ/mol, respectively. One hydrogen bond interaction was observed between amino acid residue Arg302 and acetamide moiety of **7a**. One π-aromatic interaction was found between amino acid residue Tyr306 and acetamide moiety of **7a**. Met313 and Ala88 were involved in van der Waals interaction. One π-aromatic interaction was found between amino acid residue Ala88 and xylene of **7f**. Met313 was involved in van der Waals interaction.

<table>
<tr><th>3D</th><th>2D</th></tr>
<tr><td>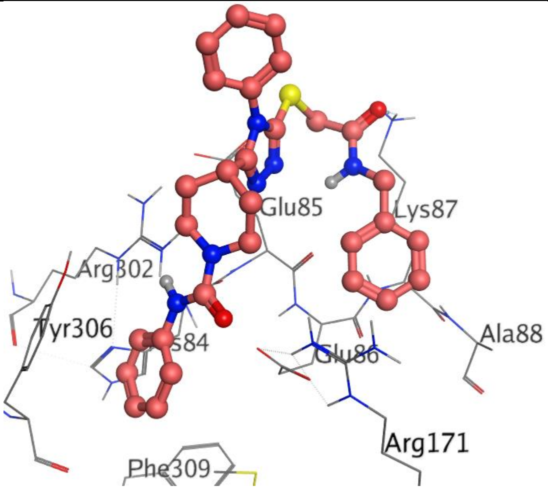

7d</td><td>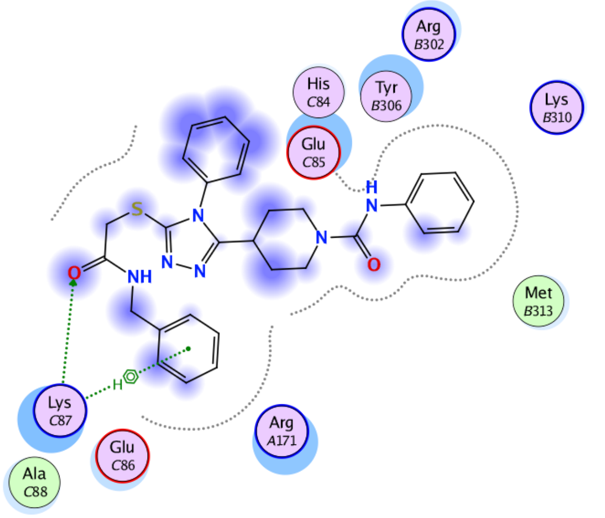
</td></tr>
<tr><td>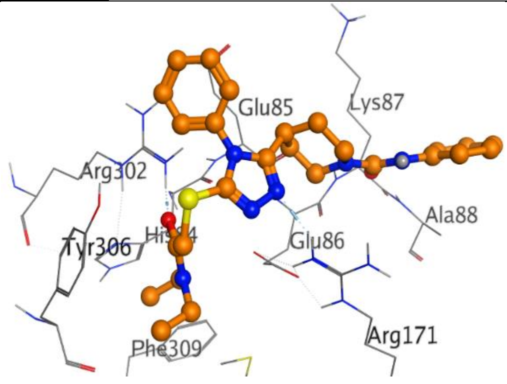

7a</td><td>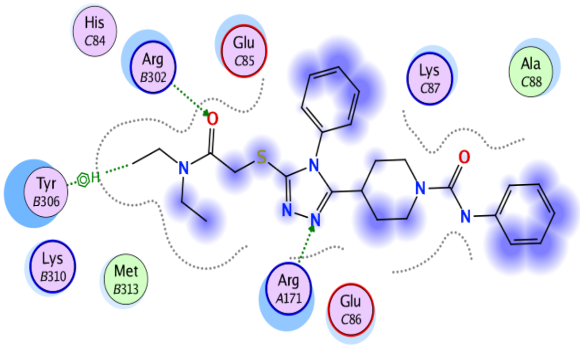
</td></tr>
</table>

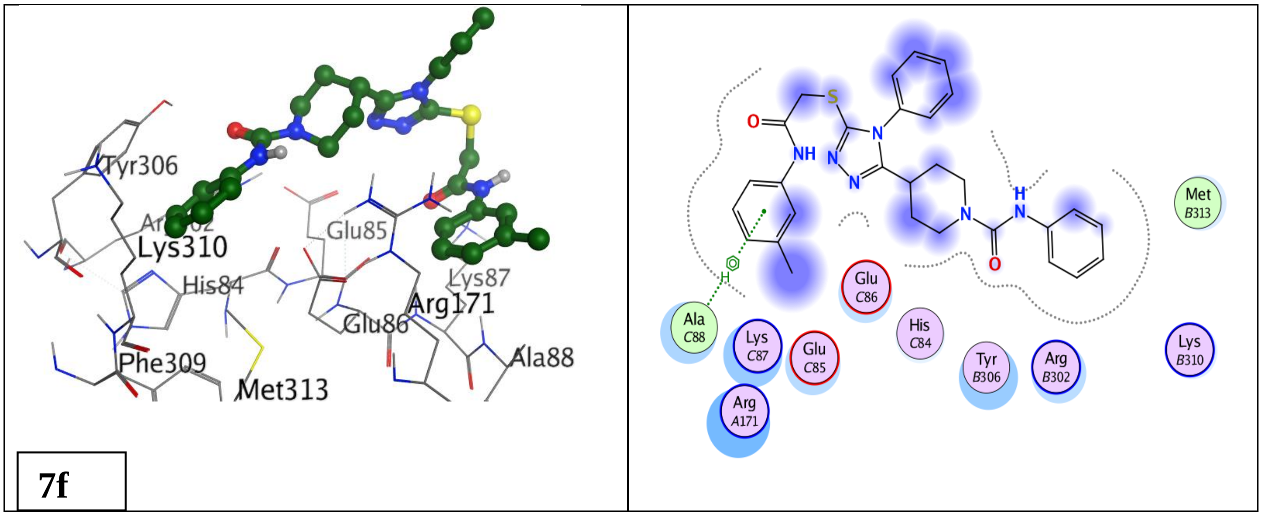

Figure S10. 3D and 2D interactions of **7d**, **7a** and **7f** with active site of NF-κB.

3.4. Molecular Dynamics Simulations

R_g is defined as the mass-weighted RMS distance of collection of atoms from their common centre of mass. R_g is considered as an important parameter while studying the stability of proteins in MD simulation studies [2]. The R_g of both proteins and their complexes is shown in **Figure S11.** As evident from the data, R_g of AKR1B10 and AKR1B10-7f complex was found to be uniform throughout the simulation duration, showing their stability. The average of R_g of AKR1B10 and AKR1B10-7f complex was obtained as 1.905 and 1.898 nm, respectively. A similar result was obtained for AKR1B1 and AKR1B1-7d complex with average R_g values as 1.915 and 1.910 nm, respectively. The R_g of all systems were stable throughout simulation period and shows that the systems did not undergo any noticeable conformational changes during the simulation [3]. SASA is another parameter to analyse the stability of proteins in MD simulations [4]. The SASA of both proteins and their complexes is shown in **Figure S12.** The SASA of AKR1B10 and AKR1B10-7f complex was roughly constant throughout the simulation. The average SASA of AKR1B10 and AKR1B10-7f complex was obtained as 151.020 and 151.470 nm^2, respectively. Similarly, average SASA of AKR1B1 and AKR1B1-7d complex was obtained as 148.602 and 147.840 nm^2, respectively.

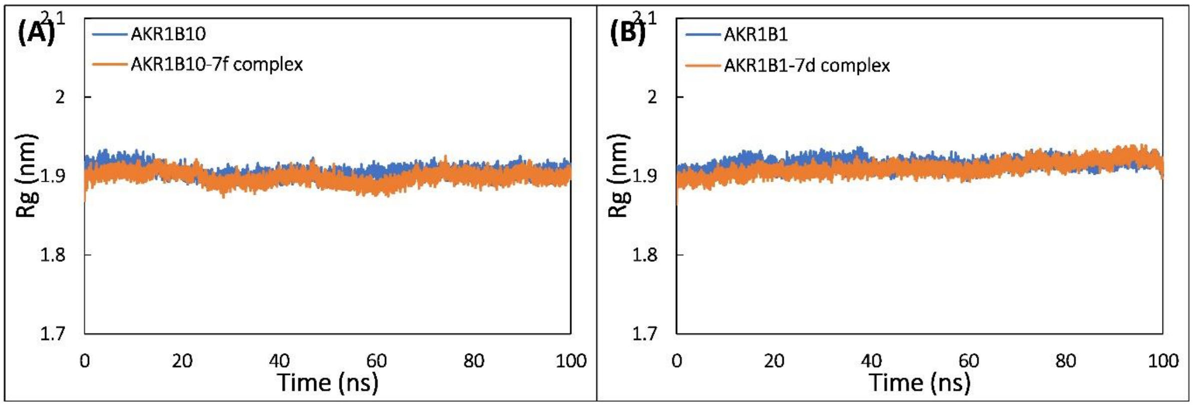

Figure S11. (A) Radius of gyration (Rg) of AKR1B10 and AKR1B10-7f complex as function of time. (B) Rg of AKR1B1 and AKR1B1-7d complex as function of time.

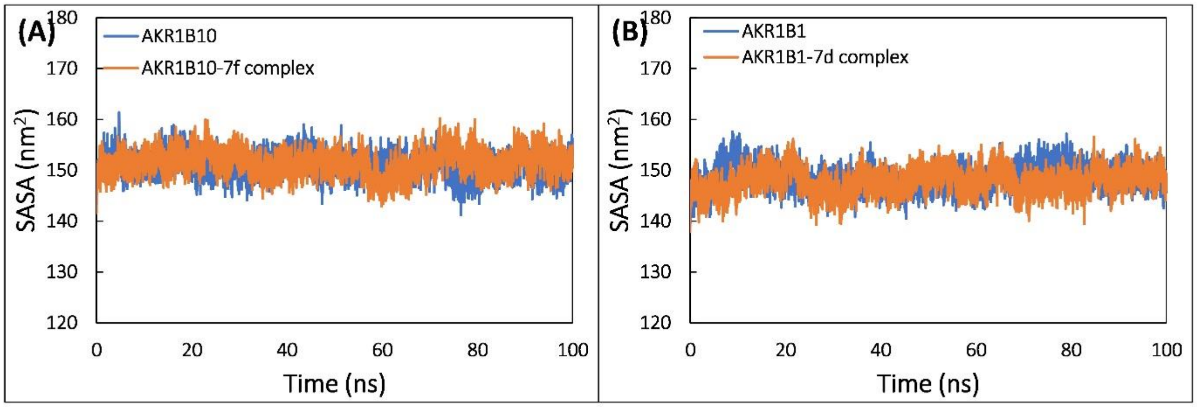

Figure S12. (A) Solvent accessible surface area (SASA) of AKR1B10 and AKR1B10-7f complex as function of time. (B) SASA of AKR1B1 and AKR1B1-7d complex as function of time.

The percentage of each secondary structural motifs in AKR1B10 and AKR1B1 in the absence and presence of their respective ligands are shown below in **Figure S13**. The percentage of coil, β-sheet, bend, turn, and α-helix in uncomplexed AKR1B10 was found to be 24.51, 12.64, 10.21, 14.27, and 31.88 respectively. There were negligible changes on these major secondary structures in presence of **7f**. Similarly, there was non-noticeable effect on the secondary

structural component of AKR1B1 after the complexation with **7d.** These results further validate the structural stability of both the complexes in aqueous environment.

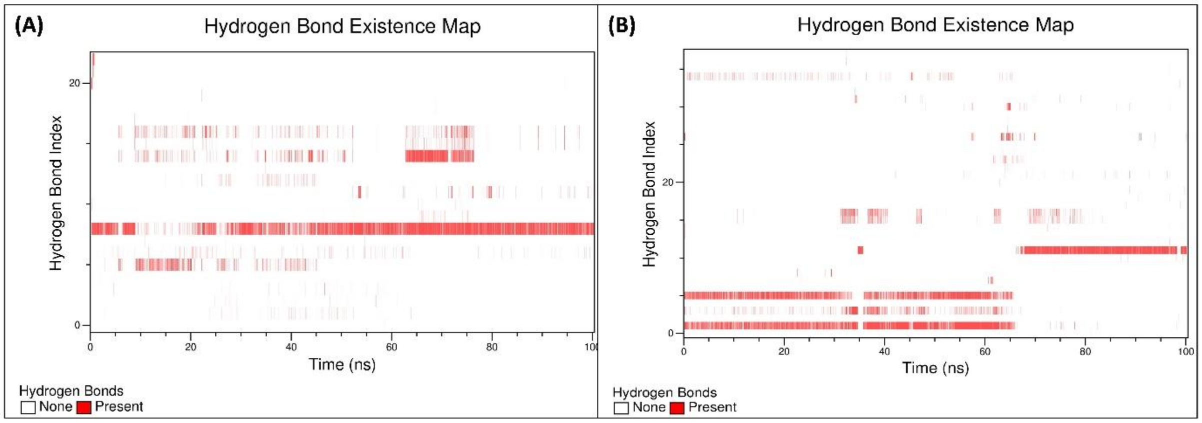

Figure S13. (A) Average secondary structural components of AKR1B10 and AKR1B10-7f complex. (B) Average secondary structural components of AKR1B1 and AKR1B1-7d complex.

All landscapes clearly show that systems reached their respective energy minima. The frame corresponding to the lowest energy were extracted from trajectories to make the Ramachandran plots shown below in (**Figure S14(B)**. The plots showed that none of residues of AKR1B10, AKR1B1, and AKR1B1-7d complex were in disallowed regions. However, only one residue was of AKR1B10-7f complex was found in disallowed regions which is negligible. Ramachandran plots again confirmed the structural stability of AKR1B10-7f complex and AKR1B1-7d complex in the aqueous environment.

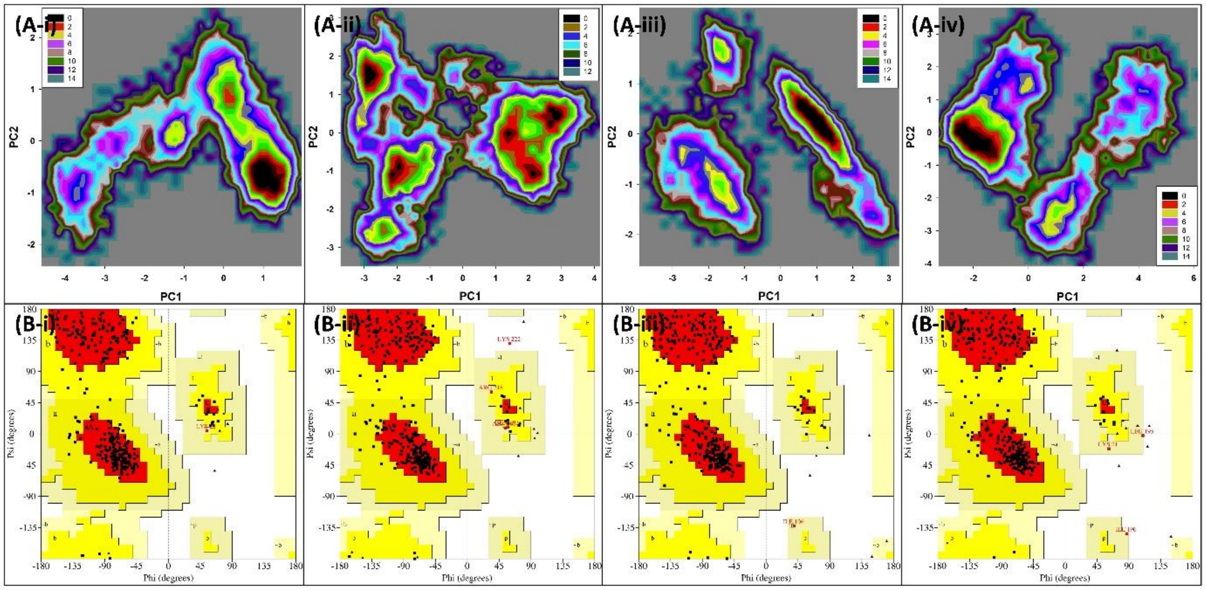

Figure S14. (A-i) Free energy landscape plot of AKR1B10. (A-ii) Free energy landscape plot of AKR1B10-7f complex. (A-iii) Free energy landscape plot of AKR1B1. (A-iv) Free energy landscape plot of AKR1B1-7d complex. (B-i) Ramachandran plot of AKR1B10. (A-ii) Ramachandran plot of AKR1B10-7f complex. (A-iii) Ramachandran plot of AKR1B1. (A-iv) Ramachandran plot of AKR1B1-7d complex.

For MM-PBSA calculations, 100 frames were taken out from 50-100 ns at uniform intervals from trajectories of the complexes. Typically, in ligand-protein interactions, the non-covalent forces are prominent. These forces include van der Waals forces, hydrophobic forces, hydrogen bonds, and electrostatic forces. These forces either contribute positively or negatively to overall binding [5]. The binding energies for interaction of **7f** and **7d** with respective proteins is enlisted in **(Table S5)**. The binding of both ligands was mostly favoured by electrostatic and van der Waals forces. Moreover, there was also contribution of solvent accessible surface area energy. On contrary, polar solvation energy impaired the binding of both the compounds. The overall binding energy for interaction of **7f** with AKR1B10 and **7d** with AKR1B1 were found to be -85.64 and -52.28 kJ/mol, respectively. From MM-PBSA calculations, the energy contribution of individual residues of both proteins was also calculated **(Table S6)**. In the binding of 7f with AKR1B10, Trp21, Lys22, Lys27, Lys78, Pro212, Leu213, Arg218, Leu229, Pro262, Lys263, Val265, and Arg269 exhibited maximum energy contribution. Likewise, Trp20, Pro211, Leu212,

Ser214, Pro215, Arg217, Leu228, and Arg268 were the major energy contributors for interaction of 7d with AKR1B1.

Table S5. Binding free energy (kJ/mol) for the interaction of **7f** with AKR1B10 and **7d** with AKR1B1 using MMBSA analysis

	**AKR1B10-7f complex**	**AKR1B1-7d complex**
**ΔE_{vdW}**	-167.68±1.32	-159.72±2.36
**ΔE_{ele}**	-72.40±1.20	-55.28±1.36
**ΔE_{PSE}**	174.56±2.00	182.04±3.48
**ΔES_{SASA}**	-20.04±0.12	-19.24±0.24
**ΔE_{BE}**	-85.64 ±1.88	-52.28±2.04

ΔE_{vdW}: van der Waals energy, ΔE_{ele}: Electrostatic energy, ΔE_{PSE}: Polar solvation energy, ΔE_{SASA}: Solvent accessible surface area energy, ΔE_{BE}: Binding energy.

Table S6. The average polar, apolar and total binding energies (kJ/mol) of the key residues

	**E_{polar}**	**E_{Apolar}**	**E_{total}**
**AKR1B10-7f complex**			
Trp21	2.216±0.128	-0.552±0.024	-3.536±0.156
Lys22	-0.436±0.120	-0.016±0.004	-2.696±0.124
Lys27	-1.056±0.160	-0.020±0.004	-3.048±0.164
Lys78	-2.292±0.288	-0.000±0.000	-4.160±0.260
Pro212	1.408±0.036	-0.112±0.004	-3.268±0.112
Leu213	1.228±0.084	-0.732±0.016	-4.324±0.160
Arg218	-5.748±0.124	-0.000±0.000	-2.912±0.140
Leu229	-0.704±0.024	-0.484±0.016	-2.860±0.112
Pro262	5.192±0.068	-0.204±0.008	-4.520±0.164
Lys263	26.328±1.044	-1.688±0.036	-22.784±0.296
Val265	1.088±0.136	-0.400±0.032	-2.120±0.172
Arg269	3.592±0.596	-1.036±0.032	-4.656±0.292
**AKR1B1-7d complex**			
Trp20	2.528±0.168	-0.544±0.016	-4.168±0.176
Pro211	1.300±0.044	-0.064±0.004	-2.268±0.096
Leu212	2.736±0.132	-0.700±0.016	-4.512±0.252
Ser214	1.732±0.252	-0.180±0.012	-2.028±0.384
Pro215	1.320±0.244	-0.444±0.032	-3.628±0.228
Arg217	-4.420±0.168	-0.004±0.000	-2.432±0.184
Leu228	0.976±0.188	-0.568±0.044	-3.596±0.280
Arg268	4.924±0.972	-0.960±0.056	-5.196±0.376

E_{polar}: Polar energy; E_{Apolar}: Apolar energy; E_{total}: Total energy.

3.6. ADMET Properties

Compound's hydrophilicity is measured by LogP, if the compound shows LogP value negative then it is hydrophilic. In **Table S7**, all the compounds are lipophilic. Lower hydrophilicities (higher LogP values), causes poor solubility and absorption [6]. LogS value manifests solubility: lower the LogS value, higher the solubility which would augment the absorption. For CNS drug-likeness, most worthwhile lipophilicity for blood–brain barrier penetration is a LogD ≤2. LogD above 4 is unacceptable for a CNS drug [7]. Topological polar surface area (TPSA) calculated for predicting oral absorption of drug-like molecules. Higher TPSA value is associated with lessened membrane permeability. Thus, lower TPSA was satisfactory for drug-likeness. For better CNS diffusion, TPSA value should be low. A derivative is said to be adequately bio-available if it has a TPSA <70 Å. Our findings showed slightly higher TPSA values, for future perspective this would be adjusted by lowering polar atoms from the synthesized compounds. Number of hydrogen bond donors (nHD) accounted the sum of all OHs and NHs while number of hydrogen bond acceptors (nHA) accounted the sum of all nitrogen and oxygen atoms without any positive charge. Optimal range for nHA 0-12 and nHD 0-7 [8].

Table S7. Physicochemical properties of the selected compounds

Code	Molecular Weight	Density	nHA	nHD	TPSA	LogS	LogP	LogD
**Control**	824.40	0.996	14	3	171.17	-4.467	3.835	2.797
**7a**	492.23	0.985	8	1	83.36	-3.893	2.934	2.784
**7b**	478.22	0.991	8	2	92.15	-3.871	2.721	3.002
**7c**	518.25	0.986	8	2	92.15	-4.716	3.918	3.466
**7d**	526.22	0.983	8	2	92.15	-4.56	3.389	3.268
**7e**	526.22	0.983	8	2	92.15	-4.745	3.898	3.291
**7f**	526.22	0.983	8	2	92.15	-5.268	4.261	3.598
**7g**	540.23	0.978	8	2	92.15	-5.172	4.397	3.639
**7h**	540.23	0.978	8	2	92.15	-5.172	4.397	3.639
**7i**	540.23	0.978	8	2	92.15	-5.162	4.478	3.54
**7j**	540.23	0.978	8	2	92.15	-4.98	4.39	3.532
**7k**	540.23	0.978	8	2	92.15	-5.091	4.438	3.585
**7l**	540.23	0.978	8	2	92.15	-4.542	3.765	3.455
**7m**	540.23	0.978	8	2	92.15	-5.706	4.837	3.83
**7n**	512.20	0.989	8	2	92.15	-4.995	3.69	3.32
**7o**	540.23	0.978	8	2	92.15	-5.091	4.438	3.585

In respect to absorption and distribution, High HIA value signifies that the compound would be better absorbed from the intestinal tract. **Table S8**, have positive values of HIV showed that all

derivatives readily cross the intestinal membrane and ultimately increase the blood plasma concentration. The calculated BBB parameter showed that most of compounds can cross BBB barrier readily, as they are mostly lipophilic in nature. Drugs should have < 90% plasma protein binding. Higher values of it have low therapeutic index. Our derivatives had greater values for plasma protein binding. When it comes to calculating the efflux by P-glycoprotein (P-gp), almost all compounds were found to be substrate and inhibitor. Volume of distribution (Vd) describes the concentration of drug in the plasma to the amount of drug in body. Optimal range is 0.04-20L/kg. All the selected derivatives fall in this optimal range [9]. The Caco-2 cell monolayer model is a surrogate in calculating the in vitro human intestinal permeability. Optimal range for Caco-2 permeability is higher than -5.15 log unit. MDCK cells used to study drug efflux and active transport, usually efflux by P-glycoprotein. Our all derivatives fall in low to medium MDCK permeability. Low permeability: <2, medium permeability: 2-20. High permeability: >20. In accordance with metabolism and excretion, all the derivatives were inhibitors of CYP1A2, CYP2C19, CYP2C9, CYP2D6 and CYP3A4. The clearance rate of drug from body has ranges; high: >15, moderate 5-15 and low <5. All the derivatives had low clearance rate except **7a** which had moderate. In terms of medicinal properties and toxicity, AMES toxicity test is implemented to know if a compound is mutagenic or not [10]. All the derivatives are not mutagenic. Synthetic accessibility score (SAscore) is to reckon the ease of synthesis of drug-like molecules. SAscore <6 is easy to synthesize. All our derivatives were easy to synthesize. The toxicity profile of the compounds was also studied. The designed compound has shown less toxicity profile based on toxicity risk assessment. The predicted results suggested that the compounds were not corrosives and irritants to eyes. They had safer profile in accordance with carcinogenicity and respiratory toxicity. Analyzing NR-AR predicts that whether a derivative activates androgen receptor or inactivates. All our derivatives were activators. Analyzing NR-AR-LBD predicts androgen receptor ligand-binding domain is activated or not. All our derivatives were activators. Analyzing NR-ER predicts about estrogen receptor activates or inactivates. All our derivatives were activators. SR-ARE is about antioxidant response element. All our derivatives were activators of SR-ARE.

Table S8. ADMET properties of phenylcarbamoylazinane-1,2,4-triazole amides derivatives (**7a-o**)

Absorption and Distribution								
	**VOLUME OF DISTRIBUTION (VD)**	**HUMAN INTESTINAL ABSORPTION (HIA)**	**CACO-2 PERMEABILITY**	**BLOOD BRAIN BARRIER (BBB)**	**PLASMA PROTEIN BINDING (PPB)**	**PGP-INHIBITOR**	**P-GLYCOPROTEIN SUBSTRATE (PGP-SUBSTRATE)**	**MDCK PERMEABILITY**
**control**	1.566	0.95	-5.708	0.172	58.96%	0.001	0.9	2e-05
**7a**	1.054	0.171	-5.391	0.956	95.11%	0.989	0.996	1.8e-05
**7b**	1.129	0.162	-5.456	0.974	94.37%	0.942	0.997	1.5e-05
**7c**	0.859	0.051	-5.409	0.829	95.66%	0.99	0.997	2.5e-05
**7d**	0.75	0.736	-5.471	0.856	96.98%	0.995	0.978	1.9e-05
**7e**	0.813	0.099	-5.414	0.611	97.92%	0.999	0.951	2.3e-05
**7f**	0.814	0.258	-5.48	0.648	98.19%	0.999	0.984	1.8e-05
**7g**	0.782	0.087	-5.415	0.603	98.21%	1.0	0.981	2.6e-05
**7h**	0.782	0.087	-5.415	0.603	98.21%	1.0	0.981	2.6e-05
**7i**	0.87	0.031	-5.414	0.517	98.42%	0.999	0.956	2.4e-05
**7j**	0.794	0.016	-5.398	0.521	98.33%	1.0	0.953	2.4e-05
**7k**	0.844	0.018	-5.398	0.488	98.48%	1.0	0.956	2.4e-05
**7l**	0.821	0.016	-5.336	0.645	98.43%	0.999	0.839	2.7e-05
**7m**	0.901	0.043	-5.468	0.601	98.66%	1.0	0.979	1.8e-05
**7n**	0.82	0.817	-5.449	0.739	97.63%	0.995	0.968	1.7e-05
**7o**	0.844	0.018	-5.398	0.488	98.48%	1.0	0.956	2.4e-05

	Metabolism					Excretion	
	**CYP1A2 inhibitor**	**CYP2C19 Inhibitor**	**CYP2C9 inhibitor**	**CYP2D6 inhibitor**	**CYP3A4 inhibitor**	**CL**	**T1/2**
**control**	0.002	0.031	0.054	0.767	0.7	1.874	0.017
**7a**	0.029	0.598	0.338	0.059	0.735	5.487	0.448
**7b**	0.043	0.692	0.755	0.21	0.889	4.262	0.559
**7c**	0.042	0.819	0.911	0.273	0.938	3.449	0.251
**7d**	0.066	0.877	0.913	0.285	0.94	3.649	0.416
**7e**	0.066	0.869	0.938	0.294	0.939	3.184	0.416
**7f**	0.091	0.894	0.947	0.519	0.949	3.748	0.34
**7g**	0.092	0.894	0.95	0.445	0.939	3.12	0.372
**7h**	0.092	0.894	0.95	0.445	0.939	3.12	0.372
**7i**	0.063	0.873	0.941	0.408	0.946	3.414	0.341
**7j**	0.058	0.855	0.933	0.299	0.946	3.508	0.38
**7k**	0.066	0.869	0.94	0.391	0.95	3.447	0.325
**7l**	0.04	0.8	0.928	0.251	0.952	2.956	0.473
**7m**	0.073	0.87	0.947	0.523	0.948	3.825	0.248
**7n**	0.088	0.877	0.947	0.378	0.914	3.423	0.395
**7o**	0.066	0.869	0.94	0.391	0.95	3.447	0.325

	Medicinal Properties		Toxicity				
	**Synthetic Accessibi**	**Lipinski Rule**	**AMES Toxicity**	**Carcinogenicity**	**Eye Corrosi**	**Eye Irritat**	**Respiratory Toxicity**

	lity Score				on	ion	
**control**	7.204	rejected	0.05	0.064	0.003	0.005	0.934
**7a**	2.368	accepted	0.013	0.382	0.003	0.014	0.688
**7b**	2.268	accepted	0.017	0.478	0.003	0.012	0.449
**7c**	2.372	accepted	0.023	0.529	0.003	0.011	0.697
**7d**	2.277	accepted	0.041	0.172	0.003	0.01	0.334
**7e**	2.283	accepted	0.14	0.607	0.003	0.013	0.667
**7f**	2.303	accepted	0.076	0.518	0.003	0.013	0.555
**7g**	2.358	accepted	0.073	0.492	0.003	0.012	0.716
**7h**	2.358	accepted	0.073	0.492	0.003	0.012	0.716
**7i**	2.361	accepted	0.222	0.612	0.003	0.013	0.543
**7j**	2.345	accepted	0.297	0.655	0.003	0.012	0.575
**7k**	2.353	accepted	0.176	0.603	0.003	0.012	0.551
**7l**	2.379	accepted	0.203	0.648	0.003	0.012	0.662
**7m**	2.347	accepted	0.2	0.621	0.003	0.013	0.526
**7n**	2.227	accepted	0.063	0.557	0.003	0.014	0.706
**7o**	2.353	accepted	0.176	0.603	0.003	0.012	0.551

TOX21 PATHWAY

	NR-AR	NR-AR-LBD	NR-ER	Antioxidant Response Element
**control**	0.024	0.002	0.495	0.064
**7a**	0.448	0.478	0.206	0.816
**7b**	0.672	0.195	0.321	0.804
**7c**	0.581	0.408	0.37	0.838
**7d**	0.738	0.586	0.343	0.826
**7e**	0.731	0.641	0.476	0.848
**7f**	0.7	0.469	0.538	0.86
**7g**	0.683	0.679	0.511	0.855
**7h**	0.683	0.679	0.511	0.855
**7i**	0.744	0.361	0.443	0.87
**7j**	0.734	0.273	0.494	0.847
**7k**	0.722	0.335	0.483	0.853
**7l**	0.781	0.167	0.362	0.815
**7m**	0.711	0.346	0.535	0.866
**7n**	0.675	0.72	0.545	0.854
**7o**	0.722	0.335	0.483	0.853

References

1. Khayami, R.; Hashemi, S.R.; Kerachian, M.A. Role of aldo-keto reductase family 1 member B1 (AKR1B1) in the cancer process and its therapeutic potential. *J. Cell. Mol. Med.* **2020**, *24*, 8890-8902.
2. Qais, F.A.; Sarwar, T.; Ahmad, I.; Khan, R.A.; Shahzad, S.A.; Husain, F.M. Glyburide inhibits non-enzymatic glycation of HSA: An approach for the management of AGEs associated diabetic complications. *Int. J. Biol. Macromol*. **2021**, *169*, 143–152.

3. Rath, B.; Qais, F.A.; Patro, R.; Mohapatra, S.; Sharma, T. Design, synthesis and molecular modeling studies of novel mesalamine linked coumarin for treatment of inflammatory bowel disease. *Bioorg. Med. Chem. Lett*. **2021**, *41*, 128029-128040.
4. Abchir, O.; Daoui, O.; Belaidi, S.; Ouassaf, M.; Qais, F.A.; ElKhattabi, S.; Belaaouad, S.; Chtita, S. Design of novel benzimidazole derivatives as potential α-amylase inhibitors using QSAR, pharmacokinetics, molecular docking, and molecular dynamics simulation studies. *J. Mol. Model.* **2022**, *28*, 1-17.
5. Siddiqui, S.; Ameen, F.; Jahan, I.; Nayeem, S.M.; Tabish, M. A comprehensive spectroscopic and computational investigation on the binding of the anti-asthmatic drug triamcinolone with serum albumin. *New J. Chem*. **2019**, *43*, 4137–4151.
6. Andleeb, H.; Hussain, M.; Ejaz, S.A.; Sevigny, J.; Farman, M.; Yasinzai, M.; Zhang, J.; Iqbal, J.; Hameed, S. Synthesis and computational studies of highly selective inhibitors of human recombinant tissue non-specific alkaline phosphatase (h-TNAP): A therapeutic target against vascular calcification..*Bioorg. Chem.* **2020**, *101*, 103999-104018.
7. Lapins, M.; Arvidsson, S.; Lampa, S.; Berg, A.; Schaal, W.; Alvarsson, J.; Spjuth, O. A confidence predictor for logD using conformal regression and a support-vector machine. *J. Cheminformatics* **2018**, *10*, 1-10.
8. Lenci, E.; Innocenti, R.; Menchi, G.; Trabocchi, A. Diversity-oriented synthesis and chemoinformatic analysis of the molecular diversity of sp3-rich morpholine peptidomimetics. *Front. Chem.* **2018**, *6*, 522.
9. Nisha, C.M.; Kumar, A.; Nair, P.; Gupta, N.; Silakari, C.; Tripathi, T.; Kumar, A. Molecular docking and in silico ADMET study reveals acylguanidine 7a as a potential inhibitor of β-secretase. *Adv. Bioinform.* **2016,** *2016,* 1-6.
10. Wang, N.N.; Dong, J.; Deng, Y.H.; Zhu, M.F.; Wen, M.; Yao, Z.J.; Lu, A.P.; Wang, J.B.; Cao, D.S. ADME properties evaluation in drug discovery: prediction of Caco-2 cell permeability using a combination of NSGA-II and boosting. *J. Chem. Inf. Model*. **2016**, *56*, 763-773.
